# Supplementary material for: B cells orchestrate tolerance to the neuromyelitis optica autoantigen AQP4
Source: Nature. 2024 Feb 21;627(8003):407–15. doi: 10.1038/s41586-024-07079-8 (PMC10937377; doi:10.1038/s41586-024-07079-8)
Supplement: Supplementary file 1 — Additional Methods for Supplementary Tables 1 and 2. [file 41586_2024_7079_MOESM1_ESM.docx]

**B cells orchestrate tolerance to the neuromyelitis optica autoantigen AQP4**

Methods to Supplementary Tables

Legends to Supplementary Tables

**Supplementary Tables**

1. **Supplementary Table 1.** Gene expression in RNAseq analysis of wild-type IgM^+^IgD^–^ thymic B cells vs. *Cd40*^–/–^ thymic B cells

2. **Supplementary Table 2.** Materials and reagents

**Methods to Supplementary Tables**

**Bulk RNA sequencing**

Total RNA was isolated from FACS sorted whole thymic B cells and thymic B cell subsets using AmpureXP beads (Beckman Coulter™). Library preparation for bulk-sequencing of poly(A)-RNA was done as described previously ^65^. Briefly, barcoded cDNA of each sample was generated with a Maxima RT polymerase (Thermo Fisher, #EP0742) using oligo-dT primer containing barcodes, unique molecular identifiers (UMIs) and an adaptor. 5‘-ends of the cDNAs were extended by a template switch oligo (TSO) and full-length cDNA was amplified with primers binding to the TSO-site and the adaptor. NEB UltraII FS kit was used to fragment cDNA. After end repair and A-tailing a TruSeq adapter was ligated and 3’-end-fragments were finally amplified using primers with Illumina P5 and P7 overhangs. In comparison to previous descriptions ^65^, the P5 and P7 sites were exchanged to allow sequencing of the cDNA in read1 and barcodes and UMIs in read2 to achieve a better cluster recognition. The library was sequenced on a NextSeq 500 (Illumina) with 65 cycles for the cDNA in read1 and 19 cycles for the barcodes and UMIs in read2. Data was processed using the published Drop-seq pipeline (v1.12) to generate sample- and gene-wise UMI tables ^66^. Reference genome (GRCm38) was used for alignment. Transcript and gene definitions were used according to GENCODE version M25.

**Bulk RNA sequencing data processing**

Raw counts of two individual sequencing runs of the same library were merged, and non-overlapping genes were dropped. Differential expression analysis was performed using the EdgeR package (edgeR_3.40.2) ^67^. After excluding lowly expressed genes (i.e. genes where a expression threshold greater than 1 count per million is not attained in at least 4 samples), the negative binomial model was fitted. The resulting P-values were adjusted for multiple testing using the FDR correction. To increase the power, we limited our analysis to genes in the serumantibodyome ^43^. Genes were considered differentially expressed if they had a less than five percent probability of being false positive (padj < 0.05). Principal component analysis (PCA) analysis was performed using the PCA function of the FactoMineR package on log counts per million (logCPM). Gene set enrichment analysis (GSEA) was performed on unfiltered DESeq2 normalized count data using the DESeq2 package (DESeq2_1.40.2) ^68^ and GSEA 4.3.2 software ^69,70^ in conjunction with MSigDB v2023.1. The interrogated gene sets were derived from the M8 collection of cell type signature gene sets. Analysis was run with permutation type phenotype and a false discovery rate (FDR) of 0.25.

**Legends to Supplementary Tables**

**Supplementary Table 1. Gene expression in RNAseq analysis of wild-type IgM^+^IgD^–^ thymic B cells vs. *Cd40*^–/–^ thymic B cells (see Fig. 3j).** Gene expression was analyzed for all genes encoding for proteins annotated as localized in the plasma membrane ^43^.

**Supplementary Table 2. Materials and Reagents.** An extensive list of all relevant materials and reagents.

**References**

43 Shome, M. *et al.* Serum autoantibodyome reveals that healthy individuals share common autoantibodies. *Cell Rep* **39**, 110873 (2022). <https://doi.org:10.1016/j.celrep.2022.110873>

65 Parekh, S., Ziegenhain, C., Vieth, B., Enard, W. & Hellmann, I. The impact of amplification on differential expression analyses by RNA-seq. *Sci Rep* **6**, 25533 (2016). <https://doi.org:10.1038/srep25533>

66 Macosko, E. Z. *et al.* Highly Parallel Genome-wide Expression Profiling of Individual Cells Using Nanoliter Droplets. *Cell* **161**, 1202-1214 (2015). <https://doi.org:10.1016/j.cell.2015.05.002>

67 McCarthy, D. J., Chen, Y. & Smyth, G. K. Differential expression analysis of multifactor RNA-Seq experiments with respect to biological variation. *Nucleic Acids Res* **40**, 4288-4297 (2012). <https://doi.org:10.1093/nar/gks042>

68 Love, M. I., Huber, W. & Anders, S. Moderated estimation of fold change and dispersion for RNA-seq data with DESeq2. *Genome Biol* **15**, 550 (2014). <https://doi.org:10.1186/s13059-014-0550-8>

69 Subramanian, A. *et al.* Gene set enrichment analysis: a knowledge-based approach for interpreting genome-wide expression profiles. *Proc Natl Acad Sci U S A* **102**, 15545-15550 (2005). <https://doi.org:10.1073/pnas.0506580102>

70 Mootha, V. K. *et al.* PGC-1alpha-responsive genes involved in oxidative phosphorylation are coordinately downregulated in human diabetes. *Nat Genet* **34**, 267-273 (2003). <https://doi.org:10.1038/ng1180>
