# Supplementary material for: B cells orchestrate tolerance to the neuromyelitis optica autoantigen AQP4
Source: Nature. 2024 Feb 21;627(8003):407–15. doi: 10.1038/s41586-024-07079-8 (PMC10937377; doi:10.1038/s41586-024-07079-8)
Supplement: Supplementary file 2 — Reporting Summary [file 41586_2024_7079_MOESM2_ESM.pdf]

nature portfolio

Corresponding author(s): Thomas Korn

Last updated by author(s): Dec 29, 2023

Reporting Summary

Nature Portfolio wishes to improve the reproducibility of the work that we publish. This form provides structure for consistency and transparency in reporting. For further information on Nature Portfolio policies, see our Editorial Policies and the Editorial Policy Checklist.

Statistics

For all statistical analyses, confirm that the following items are present in the figure legend, table legend, main text, or Methods section.

n/a	Confirmed	
☐	☒	The exact sample size (*n*) for each experimental group/condition, given as a discrete number and unit of measurement
☐	☒	A statement on whether measurements were taken from distinct samples or whether the same sample was measured repeatedly
☐	☒	The statistical test(s) used AND whether they are one- or two-sided<br>*Only common tests should be described solely by name; describe more complex techniques in the Methods section.*
☐	☒	A description of all covariates tested
☐	☒	A description of any assumptions or corrections, such as tests of normality and adjustment for multiple comparisons
☐	☒	A full description of the statistical parameters including central tendency (e.g. means) or other basic estimates (e.g. regression coefficient) AND variation (e.g. standard deviation) or associated estimates of uncertainty (e.g. confidence intervals)
☐	☒	For null hypothesis testing, the test statistic (e.g. *F*, *t*, *r*) with confidence intervals, effect sizes, degrees of freedom and *P* value noted<br>*Give P values as exact values whenever suitable.*
☒	☐	For Bayesian analysis, information on the choice of priors and Markov chain Monte Carlo settings
☐	☒	For hierarchical and complex designs, identification of the appropriate level for tests and full reporting of outcomes
☐	☒	Estimates of effect sizes (e.g. Cohen's *d*, Pearson's *r*), indicating how they were calculated

Our web collection on statistics for biologists contains articles on many of the points above.

Software and code

Policy information about availability of computer code

Data collection	BD FACSDIVA (v.8.0.1); CytExpert (v.2.5.0.77); NextSeq 500 (Illumina); Leica Applications Suite X (v.3.5.6.21594); Leica LCS (v.2.6.1.5173); NIS Elements AR (v.5.20.00); Zeiss Axio Imager Z2 (ZEN 3.6); Leica TCS SP8 confocal microscope with HC PL APO CS2 40x/1.30NA objective; Imaris 9.7 software (Oxford Instruments); 3DHistech Pannoramic Flash II with Visopharm software (v2023.1).
Data analysis	Cell Ranger (v.7.1.0); Python (v.3.9.16); Scanpy (v.1.9.2); Scrublet (v.0.2.3); Velocyto (v.0.17.17); ScVelo (v.0.2.5); UnitVelo (v.0.2.5.2); QuPath (v.0.3.2); Prism (v.10.9.0); FlowJo (v.10.5.1); Adobe Illustrator 2022 (v.26.0.1); Adobe Photoshop CS6 (v.13.0); R (v.4.2.2); RStudio (v2023.06.01); Drop-seq pipeline (v1.12); edgeR (v.3.40.2); limma (v3.54.0); FactoMineR (v.2.6); factoextra (v.1.0.7); ggplot2 (v2.3.4); leidenalg package (0.9.1), DESeq2 (v1.40.2); GSEA software (v4.3.2).

For manuscripts utilizing custom algorithms or software that are central to the research but not yet described in published literature, software must be made available to editors and reviewers. We strongly encourage code deposition in a community repository (e.g. GitHub). See the Nature Portfolio guidelines for submitting code & software for further information.

Data

Policy information about availability of data

All manuscripts must include a data availability statement. This statement should provide the following information, where applicable:

- Accession codes, unique identifiers, or web links for publicly available datasets
- A description of any restrictions on data availability
- For clinical datasets or third party data, please ensure that the statement adheres to our policy

Reference genome (GRCm38) was used for alignment of bulk RNAseq data. Transcript and gene definitions were used according to GENCODE version M25. Single cell RNAseq reads were aligned to mouse reference genome mm10-2020 as provided by 10x genomics. NGS raw data, processed gene expression data and spliced/unspliced count data have been deposited in the GEO repository under the accession number GSE234188 (scRNAseq mouse) and GSE244363 (bulkRNAseq mouse). No custom algorithms were used in this study. Public thymic scRNAseq data was downloaded from https://zenodo.org/record/5500511 (DOI: 10.5281/zenodo.5500511)

Research involving human participants, their data, or biological material

Policy information about studies with human participants or human data. See also policy information about sex, gender (identity/presentation), and sexual orientation and race, ethnicity and racism.

Reporting on sex and gender	This information has not been collected due to its irrelevance for the study.
Reporting on race, ethnicity, or other socially relevant groupings	This information has not been collected due to its irrelevance for the study.
Population characteristics	This information has not been collected due to its irrelevance for the study.
Recruitment	Human tonsillar tissue was obtained from routine tonsillectomies by the Department of Otorhinolaryngology of the University Hospital “Klinikum rechts der Isar” of the Technical University of Munich (School of Medicine, Germany) with patient’s informed consent.<br>Human newborn thymic tissue was obtained from Andreas Büttner, University of Rostock (Approval by the local ethics committee).
Ethics oversight	The study was approved by the local ethics committee of the University of Rostock (A 2023-0038).

Note that full information on the approval of the study protocol must also be provided in the manuscript.

Field-specific reporting

Please select the one below that is the best fit for your research. If you are not sure, read the appropriate sections before making your selection.

☒ Life sciences ☐ Behavioural & social sciences ☐ Ecological, evolutionary & environmental sciences

For a reference copy of the document with all sections, see nature.com/documents/nr-reporting-summary-flat.pdf

Life sciences study design

All studies must disclose on these points even when the disclosure is negative.

Sample size	This study is purely explorative. No statistical methods were used to predetermine the sample size. Sample sizes were determined to be sufficient based on established standards for explorative studies in the field. Importantly, whenever statistical analysis was applied the sample size was ≥3 biological replicates per group.
Data exclusions	Figure 3 scRNAseq data: For quality control, cells with more than 15% mitochondrial gene counts were excluded as well as cells with more than 2% hemoglobin gene counts and cells with less than 5% ribosomal gene counts. Cells with less than 200 detected genes and cells with more than 1E4 counts per cell were also removed. Doublet exclusion was performed using scrublet.<br><br>Figure 3 bulkRNA seq data: For quality control, genes with expression values lower than 1 count per million in at least 4 samples were excluded. To increase our power, we limited our analysis to genes in the serumantibodyome, as explained in the Methods section.
Replication	Biological replicates were used in this study to ensure reproducibility. Most of the experiments were repeated at least twice as stated in the figure legends. All attempts at replication were successful.
Randomization	Randomization was not necessary for this study since there was no particular intervention (e.g. treatment) planned. Whenever possible littermate controls were used and equal number of mice were allocated to different groups within one cage to rule out cage effects.
Blinding	For mice experiments blinding during data collecting was usually not possible due to required cage labeling. However, scoring of EAE mice was occasionally performed by members of the staff who were not familiar with the experimental design. Furthermore, data analysis used in this

study was strictly quantitative (and not subjective) and therefore blinding was not necessary.
For the human data, samples were provided to us in a blinded manner by different collaborators without any patient's individual information.

Reporting for specific materials, systems and methods

We require information from authors about some types of materials, experimental systems and methods used in many studies. Here, indicate whether each material, system or method listed is relevant to your study. If you are not sure if a list item applies to your research, read the appropriate section before selecting a response.

Materials & experimental systems

n/a	Involved in the study	
☐	☒	Antibodies
☐	☒	Eukaryotic cell lines
☒	☐	Palaeontology and archaeology
☐	☒	Animals and other organisms
☒	☐	Clinical data
☒	☐	Dual use research of concern
☒	☐	Plants

Methods

n/a	Involved in the study	
☒	☐	ChIP-seq
☐	☒	Flow cytometry
☒	☐	MRI-based neuroimaging

Antibodies

Antibodies used

The following antibodies were used for flow cytometry:
Arm. Hamster BV421 CD95 (Jo2, BD Biosciences, #562633, 1:300, RRID: AB_2737690)
Arm. Hamster PE CD95 (Jo2, BD Biosciences, #554258, 1:300, RRID: AB_395330)
Arm. Hamster FITC CD3e (145-2C11, eBioscience, #11-0031, 1:300, RRID: AB_464882)
Arm. Hamster FITC TCRß (H57-597, BD, #553171, 1:300, RRID: AB_394683)
Arm. Hamster PE-Cy7 CD11c (N418, BioLegend, #117318, 1:300, RRID: AB_493569)
Mouse Alexa Fluor 488 IgD (IA6-2, Biolegend, #348216, 1:200, RRID: AB_11150595)
Mouse APC CD10 (97C5, Miltenyi, #130-119-675, 1:100, RRID: AB_2660858)
Mouse APC-CD19 (SJ25C1, Biolegend, #363005, 1:300, RRID: AB_2564127)
Mouse BV421 Bcl6 (K112-91, BD Biosciences, #563363, 1:200, RRID: AB_2738159)
Mouse BV421 CD2 (RPA-2.10, BD Biossciences, #562667, 1:300, RRID: AB_2737695)
Mouse BV510 CD14 (M5E2, BioLegend, #301842, 1:300, RRID: AB_2561379)
Mouse BV421 CD19 (HIB19, BioLegend, #302234, 1:200, RRID: AB_11142678)
Mouse BV510 CD27 (M-T271, Biolegend, #356420, 1:200, RRID: AB_2562603)
Mouse BV421 CD45.1 (A20, BioLegend, #110732, 1:300, RRID: AB_10896425)
Mouse BV421 NK1.1 (PK136, eBioscience, #48-5941, 1:300, RRID: AB_2043877)
Mouse BV510 NK1.1 (PK136, BioLegend, #108738, 1:300, RRID: AB_2562216)
Mouse BV711 CD38 (HIT2, BD Biosciences, #563965, 1:100, RRID: AB_2738516)
Mouse BV786 CD45.2 (104, BD Biosciences, #563686, 1:300, RRID: AB_2738375)
Mouse BV786 RORyt (Q31-378 , BD Biosciences, #564723, 1:100, RRID: AB_2738916)
Mouse FITC IgM (AF6-78, BD Biosciences, #553520, 1:300, RRID: AB_394901)
Mouse FITC NK1.1 (PK136, eBioscience, #11-5941, 1:300, RRID: AB_465319)
Mouse PE CD3 (UCHT1, Beckman Coulter, #IM1282U, 1:200, RRID: AB_467059)
Mouse PE-Cy7 T-bet (4B10, BioLegend, #644824, 1:200, RRID: AB_2561760)
Mouse PE-Cy7-CD3 (SK7, BD Biosciences, #341111, 1:300, RRID: AB_10596664)
Mouse PerCP/Cy5.5-CD27 (O323, BioLegend, #302819, 1:300, RRID: AB_11218994)
Goat AF488 IgG (H+L) (ThermoFisher, #A-11029, 1:100, RRID: AB_2534088)
Rat CD16/CD32 (2.4G2, BD Biosciences, #553142, 1:100, RRID: AB_394657)
Rat AF488 Foxp3 (FJK-16s, eBioscience, #53-5773, 1:200, RRID: AB_763537)
Rat AF647 CD5 (53-7.3, BioLegend, #100614, 1:300, RRID: AB_493168)
Rat APC CD19 (1D3, BD Biosciences, #550992, 1:300, RRID: AB_398483)
Rat APC IgD (11-26c.2a, BioLegend, #405714, 1:300, RRID: AB_10645480)
Rat APC Ly6A/E (D7, BioLegend, #122512, 1:300, RRID: AB_756196)
Rat APC MHC-II (M5/114.15.2, eBioscience, #17-5321, 1:300, RRID: AB_469454)
Rat APC PD-1 (29F.1A12, BioLegend, #135210, 1:300, RRID: AB_2251944)
Rat APC-R700 B220 (RA3-6B2, BioLegend, #103232, 1:300, RRID: AB_493716)
Rat BV421 B220 (RA3-6B2, BioLegend, #103227, 1:300, RRID: AB_492877)
Rat BV421 EpCAM (G8.8, BioLegend, #118225, 1:300, RRID: AB_2563983)
Rat BV510 CD11b (M1/70, BioLegend, #101263, 1:300, RRID: AB_2561390)
Rat BV510 CD19 (6D5, BioLegend, #115546, 1:300, RRID: AB_2562136)
Rat BV510 F4/80 (BM8, BioLegend, #123135, 1:300, RRID: AB_2562622)
Rat BV786 CD19 (6D5, Biolegend, #115543, 1:300, RRID: AB_11218994)
Rat BV786 Vb6 (RR4-7, BD Biosciences, #744595, 1:200, RRID: AB_2742344)
Rat FITC CD11b (M1/70, BioLegend, #101205, 1:300, RRID: AB_312788)
Rat FITC CD4 (GK1.5, BD Biosciences, #553729, 1:500, RRID: AB_395013)
Rat FITC F4/80 (BM8, abcam, #ab60343, 1:300, RRID: AB_2637191)
Rat PE CD19 (1D3, BD Biosciences, #557399, 1:300, RRID: AB_395050)
Rat PE CD8a (53-6.7, BD Bioscience, #553033, 1:300, RRID: AB_394571)

Rat PE Ly6A/E (D7, Thermo, #12-5981-82, 1:300, RRID: AB_466086)
Rat PE GL7 (GL7, BD Biosciences, #561530, 1:300, RRID: AB_10715834)
Rat PE-Cy7 B220 (RA3-6B2, BioLegend, #103222, 1:300, RRID: AB_313004)
Rat PE-Cy7 CD21 (7E9, BioLegend, #123420, 1:300, RRID: AB_1953276)
Rat PerCP CD19 (6D5, BioLegend, #115532, 1:300, RRID: AB_893278)
Rat PerCP-Cy5.5 B220 (RA3-6B2, BioLegend, #103236, 1:300, RRID: AB_893356)
Rat PerCP-eF710 CD4 (RM4-5, eBioscience, #46-0042, 1:500, RRID: AB_1834431)

The following antibodies were used in cell hashing: TotalSeq-C anti-mouse Hashtag 1 to 9 (M1/42; 39-F11, Biolegend, #155861, #155863, #155865, #155867 ,#155869, #155871, #155873, #155875, #155877). All antibodies were diluted 1:100.

The following antibodies were used in T cell cultures:
Mouse anti-CD3e (500A2, eBioscience, #16-0033-86, 1:2000, RRID: AB_842783) and
Rat anti-CD40 (FGK45, BioXCell, BE0016-2, AB_1107601)
AffiniPure F(ab')$_2$ Fragment goat anti-IgG (H+L) (Jackson Immuno Research, 109-006-088, AB_2337549)
AffiniPure F(ab')$_2$ Fragment goat anti-IgG + anti-IgM (H+L) (Jackson Immuno Research, 115-006-068, AB_2338471)

The following antibodies were used in immunohistochemistry:
Mouse CD20 (L26, Dako, #M0755, 1:500, validated by manufacturer)
Mouse GFAP (G-A-5, Sigma-Aldrich, #G6171, 1:400, validated by manufacturer)
Rabbit AQP4 (Sigma, #HPA014784, 1:2000, validated by manufacturer)
Rabbit Bcl6 (D65C10, Cell signaling, #5650, 1:100, validated by manufacturer)
Rabbit CD19 (D4V4B, Cell signaling, #90176, 1:400, validated by manufacturer)
Rabbit EpCAM (abcam, #ab71916, 1:200, validated by manufacturer)
Rat B220 (RA3-6B2, BD Biosciences, #550286, RRID: AB_394619)
Rat CD19 (6OMP31, eBiosciences, #14-0194-80, 1:200, RRID: AB_2637171)
Rat CD45 (30-F11, ThermoFisher, #14-0451--82, 1:500, RRID: AB_467251)
Donkey polyclonal secondary antibody to mouse IgG (H&L), preabsorbed, Alexa Fluor 568 (Thermo, #A10037, 1:2000, RRID: AB_2534013)
Donkey polyclonal secondary antibody to rabbit IgG (H&L), preabsorbed, Alexa Fluor 488 (Thermo, #A21206, 1:2000, RRID: AB_2535792)
Donkey polyclonal secondary antibody to rabbit IgG (H&L), preabsorbed, Alexa Fluor 647 (Life, #A31573, 1:200, RRID: AB_2536183)
Goat polyclonal secondary antibody to rabbit IgG (H&L), preabsorbed, Alexa Fluor 647 (Abcam, #ab150087, 1:400, validated by manufacturer)
Goat polyclonal secondary antibody to rat IgG, conjugated to horseradish peroxidase (HRP) (Vector, #MP-7444, 1:3000, validated by manufacturer).

Validation

All antibodies are commercially available, widely used and validated by the manufacturer for the same application and species as used in this study. Please find details about validation in the RRID registry (https://scicrunch.org/resources/Antibodies/search?l=&q=%2A, RRID identifiers are listed above) and the manufacturer's website. In addition, control stainings were performed with cell types known to express or lack the relevant antigens.

Eukaryotic cell lines

Policy information about cell lines and Sex and Gender in Research

Cell line source(s)

The T cell hybridoma cell line A5 was kindly provided by L. Klein (LMU Munich, Germany) as described in the method section.
The fibroblastic YKL cell line was kindly provided by M. Schmidt-Supprian (TU Munich, Gemany) as described in the method section.
The packing cell line Plat-E was kindly provided by D. Busch (TU Munich, Germany) as described in the method section.
Various manufacturers offer these cells.

Authentication

Cell line was authenticated prior to receipt by the commercial vendor using the STR method.

Mycoplasma contamination

Cells were not tested for mycoplasma after receipt.

Commonly misidentified lines
(See ICLAC register)

No commonly misidentified cell lines were used.

Animals and other research organisms

Policy information about studies involving animals; ARRIVE guidelines recommended for reporting animal research, and Sex and Gender in Research

Laboratory animals

For animal experiments sex- and age-matched female and male mice on C57BL/6J background were used.
The mice used in studies investigating thymi were between 6 and 10 weeks old, while those used for immunization were between 10 and 18 weeks old.

C57BL/6J, Aire-flox/flox, Rag1-/-, Cd40–/– and TCRa–/– mice were obtained from The Jackson Laboratory.
Aqp4–/– mice were kindly provided by A. Verkman (University of California, San Francisco UCSF).
Aqp4flox/flox mice were kindly provided by O. P. Ottersen (University of Oslo).
Foxn1-Cre mice were kindly provided by L. Klein (Ludwig Maximillians University, Munich).
Mb1-Cre mice were kindly provided by M. Schmidt-Supprian (Technical University of Munich).
DEREG mice were kindly provided by Tim Sparwasser (Johannes Guttenberg University of Mainz).

	Mice were housed with a dark/light cycle of 12 hours, a temperature of 20-24°C, and a humidity of 45-60%.
Wild animals	The study did not involve wild animals.
Reporting on sex	Male and female mice were used equally for all experiments.
Field-collected samples	The study did not involve samples collected form the field.
Ethics oversight	Experimental procedures were approved and performed according to the the Bavarian state authorities as indicated in the method section.

Note that full information on the approval of the study protocol must also be provided in the manuscript.

Flow Cytometry

Plots

Confirm that:

☒ The axis labels state the marker and fluorochrome used (e.g. CD4-FITC).

☒ The axis scales are clearly visible. Include numbers along axes only for bottom left plot of group (a 'group' is an analysis of identical markers).

☒ All plots are contour plots with outliers or pseudocolor plots.

☒ A numerical value for number of cells or percentage (with statistics) is provided.

Methodology

Sample preparation	Sample preparation as described in the methods.
Instrument	CytoFLEX (Beckman Coulter); FACS Aria III (BD Biosciences)
Software	BD FACSDIVA (v.8.0.1); CytExpert (v.2.3.1.22); FlowJo (v10.9.0)
Cell population abundance	Cell sorting was performed with the strictest purity setting (4-Way Purity), ensuring a purity of >95%.
Gating strategy	See flow cytometric gating strategy as clarified in the Extended data figures. Debris and dead cells were gated out based on a distinctive FSC and SSC gate specific for lymphocytes as is common practice. Singlets were gated based on FSC-A/FSC-H and SSC-A/SSC-H. Dump channel exclusion of dead cells (Aqua LiveDead positive), CD11b, NK1.1, F4/80 and either CD19 or CD3 as further clarified in the method section.

☒ Tick this box to confirm that a figure exemplifying the gating strategy is provided in the Supplementary Information.
