# Supplementary material for: B cells orchestrate tolerance to the neuromyelitis optica autoantigen AQP4
Source: Nature. 2024 Feb 21;627(8003):407–15. doi: 10.1038/s41586-024-07079-8 (PMC10937377; doi:10.1038/s41586-024-07079-8)
Supplement: Supplementary file 4 — Materials and reagents. [file 41586_2024_7079_MOESM4_ESM.pdf]

Supplementary Table 2

Taqman probes

Gene	Assay ID	Species	Company	Catalog #
AQP4	Mm00802129_m1	Mouse	Thermo Fisher	4351372
Aire	Mm00477461_m1	Mouse	Thermo Fisher	4331182
GAPDH	Mm99999915_g1	Mouse	Thermo Fisher	4331182
MOG	MM01279062_m1	Mouse	Thermo Fisher	4331182
AQP4	Hs00242341_m1	Human	Thermo Fisher	4331182
GAPDH	Hs02786624_g1	Human	Thermo Fisher	4351370

Antibodies for flow cytometry

Marker	Clone	Species	Fluoro-phore	Company	Catalog#	Titration	RRID
B220	RA3-6B2	Rat	APC-R700	BioLegend	103232	1:300	AB_493716
B220	RA3-6B2	Rat	BV421	BioLegend	103227	1:300	AB_492877
B220	RA3-6B2	Rat	PE-Cy7	BioLegend	103222	1:300	AB_313004
B220	RA3-6B2	Rat	PerCP-Cy5.5	BioLegend	103236	1:300	AB_893356
Bcl6	K112-91	Mouse	BV421	BD	563363	1:200	AB_2738159
CD2	RPA-2	Mouse	BV421	BD	562667	1:300	AB_2737695
CD3e	145-2C11	Arm. Hamster	FITC	eBioscience	11-0031	1:300	AB_464882
CD3e	UCHT1	Mouse	PE	Beckman Coulter	IM1282U	1:200	AB_467059
CD3e	SK7	Mouse	PE-Cy7	BD	341111	1:300	AB_10596664

CD4	GK1.5	Rat	FITC	BD	553729	1:500	AB_395013
CD4	RM4-5	Rat	PerCP-eF710	eBioscience	46-0042	1:500	AB_1834431
CD5	53-7.3	Rat	AF647	BioLegend	100614	1:300	AB_493168
CD8a	53-6.7	Rat	PE	BD	553033	1:300	AB_394571
CD10	97C5	Mouse	APC	Miltenyi	130-119-675	1:100	AB_2660858
CD11b	M1/70	Rat	BV510	BioLegend	101263	1:300	AB_2561390
CD11b	M1/70	Rat	FITC	BioLegend	101205	1:300	AB_312788
CD11c	N418	Arm. Hamster	PE-Cy7	BioLegend	117318	1:300	AB_493569
CD14	M5E2	Mouse	BV510	BioLegend	301842	1:300	AB_2561379
CD16/ CD32	2.4G2	Rat	-	BD	553142	1:100	AB_394657
CD19	1D3	Rat	APC	BD	550992	1:300	AB_398483
CD19	1D3	Rat	PE	BD	557399	1:300	AB_395050
CD19	6D5	Rat	BV510	BioLegend	115546	1:300	AB_2562136
CD19	6D5	Rat	BV786	BioLegend	115543	1:300	AB_11218994
CD19	6D5	Rat	PerCP	BioLegend	115532	1:300	AB_893278
CD19	HIB19	Mouse	BV421	BioLegend	302234	1:200	AB_11142678
CD19	SJ25C1	Mouse	APC	BioLegend	363005	1:300	AB_2564127
CD21	7E9	Rat	PE-Cy7	BioLegend	123420	1:300	AB_1953276
CD27	M-T271	Mouse	BV510	BioLegend	356420	1:200	AB_2562603
CD27	O323	Mouse	PerCP-C5.5	BioLegend	302819	1:30	AB_11218994
CD38	HIT2	Mouse	BV711	BD	563965	1:100	AB_2738516
CD45.1	A20	Mouse	BV421	BioLegend	110732	1:300	AB_10896425
CD45.2	104	Mouse	BV786	BD	563686	1:300	AB_2738375

CD95	Jo2	Arm. Hamster	BV421	BD	562633	1:300	AB_2737690
CD95	Jo2	Arm. Hamster	PE	BD	554258	1:300	AB_464882
EpCAM	G8.8	Rat	BV421	BioLegend	118225	1:300	AB_2563983
F4/80	BM8	Rat	BV510	BioLegend	123135	1:300	AB_2562622
F4/80	BM8	Rat	FITC	abcam	ab60343	1:300	AB_2637191
Foxp3	FJK-16s	Rat	AF488	eBioscience	53-5773	1:200	AB_763537
GL7	GL7	Rat	PE-Cy7	BD	561530	1:300	AB_10715834
IgD	IA6-2	Mouse	AF488	BioLegend	348216	1:200	AB_11150595
IgD	11-26c.2a	Rat	APC	BioLegend	405714	1:300	AB_10645480
IgG (H+L)	/	Goat	AF488	Thermo	A-11029	1:100	AB_2534088
IgM	AF6-78	Mouse	FITC	BD	553520	1:300	AB_394901
Ly6A/E	D7	Rat	APC	BioLegend	122512,	1:300	AB_756196
Ly6A/E	D7	Rat	APC	Thermo	12-5981-82	1:300	AB_466086
MHC-II	M5/114.15.2	Rat	APC	eBioscience	17-5321	1:300	AB_469454
NK1.1	PK136	Mouse	BV421	eBioscience	48-5941	1:300	AB_2043877
NK1.1	PK136	Mouse	BV510	BioLegend	108738	1:300	AB_2562216
NK1.1	PK136	Mouse	FITC	eBioscience	11-5941	1:300	AB_465319
PD-1	29F.1A12	Rat	APC	BioLegend	135210	1:300	AB_2251944
RORyt	Q31-378	Mouse	BV786	BD	564723	1:100	AB_2738916
T-bet	4B10	Mouse	PE-Cy7	BioLegend	644824	1:200	AB_2561760
TCRß	H57-597	Arm. Hamster	FITC	BD	553171	1:300	AB_394683
Vβ6	RR4-7	Rat	BV786	BD	744595	1:200	AB_2742344

Antibodies for immunohistochemistry

Marker	Clone	Species	Company	Catalog#	Titration	Validation
AQP4	Polyclonal	Rat	Sigma	HPA014784	1:2000	Manufacturer
B220	RA3-6B2	Rat	BD	550286	1:300	AB_394619
Bcl6	D65C10	Rabbit	Cell signaling	5650	1:100	Manufacturer
CD19	D4V4B	Rabbit	Cell signaling	90176	1:400	Manufacturer
CD19	6OMP31	Rat	eBioscience	14-0194-80	1:200	AB_2637171
CD20	L26	Mouse	Dako	M0755	1:500	Manufacturer
CD45	30-F11	Rat	Thermo	14-0451-82	1:500	AB_467251
EpCAM	Polyclonal	Rabbit	abcam	ab71916	1:200	Manufacturer
GFAP	G-A-5	Mouse	Sigma	G6171	1:400	Manufacturer
IgG (H+L) AF488	Polyclonal	Donkey	Thermo	A21206	1:2000	AB_2535792
IgG (H+L) AF568	Polyclonal	Donkey	Thermo	A10037	1:2000	AB_2534013
IgG (H+L) AF647	Polyclonal	Goat	abcam	ab150087	1:500	Manufacturer
IgG (H+L) AF647	Polyclonal	Donkey	Life	A31573	1:200	AB_2536183
IgG HRP	Polyclonal	Goat	Vector	MP-7444	1:3000	Manufacturer
